# Change in Eosinophil Count in Patients with Heart Failure Treated with Anakinra

**DOI:** 10.3390/cells12081129

**Published:** 2023-04-11

**Authors:** Michele Golino, Francesco Moroni, Marco Giuseppe Del Buono, Justin M. Canada, Azita H. Talasaz, Sebastian Piñel, James Mbualungu, Alessandra Vecchiè, Ai-Chen (Jane) Ho, Georgia K. Thomas, Salvatore Carbone, Hayley E. Billingsley, Jeremy Turlington, Roshanak Markley, Cory Trankle, Roberto De Ponti, Benjamin Van Tassell, Antonio Abbate

**Affiliations:** 1 Pauley Heart Center, Virginia Commonwealth University, Richmond, VA 23284, USA; micheleg1390@gmail.com (M.G.); frnmoroni@gmail.com (F.M.);; 2 Department of Medicine and Surgery, University of Insubria, 21100 Varese, Italy; 3 Department of Internal Medicine, University of Virginia, Charlottesville, VA 22904, USA; 4 Department of Medicine, Università Milano-Bicocca, 20126 Milan, Italy; 5 Department of Cardiovascular Medicine, Fondazione Policlinico Universitario A. Gemelli IRCCS, 00168 Rome, Italy; 6 Department of Pharmacotherapy and Outcome Sciences, Virginia Commonwealth University, Richmond, VA 23284, USA; 7 Robert M. Berne Cardiovascular Research Center, Division of Cardiology—Heart and Vascular Center, University of Virginia, Charlottesville, VA 22904, USA; 8 Medicina Generale 1, Medical Center, Department of Internal Medicine, Ospedale di Circolo e Fondazione Macchi, ASST Sette Laghi, 21100 Varese, Italy; 9 Department of Clinical & Administrative Sciences, School of Pharmacy, Notre Dame of Maryland University, Baltimore, MD 21210, USA; 10 Department of Kinesiology & Health Sciences, College of Humanities & Sciences, Virginia Commonwealth University, Richmond, VA 23284, USA

**Keywords:** eosinophils, heart failure, interleukin-1, interleukin-1 receptor antagonist protein, injection site reaction, cardiorespiratory fitness, exercise test

## Abstract

Background: Interleukin-1 blockade with anakinra leads to a transient increase in eosinophil blood count (eosinophils) in patients with acute myocardial infarction. We aimed to investigate the effect of anakinra on changes in eosinophils in patients with heart failure (HF) and their correlation with cardiorespiratory fitness (CRF). Methods: We measured eosinophils in 64 patients with HF (50% females), 55 (51–63) years of age, before and after treatment, and, in a subset of 41 patients, also after treatment cessation. We also evaluated CRF, measuring peak oxygen consumption (VO_2_) with a treadmill test. Results: Treatment with anakinra significantly and transiently increased eosinophils, from 0.2 [0.1–0.3] to 0.3 [0.1–0.4] × 10^3^ cells/µL (*p* < 0.001) and from 0.3 [0.2–0.5] to 0.2 [0.1–0.3] × 10^3^ cells/µL, with suspension (*p* < 0.001). Changes in eosinophils correlated with the changes in peak VO_2_ (Spearman’s Rho = +0.228, *p* = 0.020). Eosinophils were higher in patients with injection site reactions (ISR) (*n* = 8, 13%; 0.5 [0.4–0.6] vs. 0.2 [0.1–0.4] × 10^3^ cells/µL, *p* = 0.023), who also showed a greater increase in peak VO_2_ (3.0 [0.9–4.3] vs. 0.3 [−0.6–1.8] mLO_2_·kg^−1^·min^−1^, *p* = 0.015). Conclusion: Patients with HF treated with anakinra experience a transient increase in eosinophils, which is associated with ISR and a greater improvement in peak VO_2_.

## 1. Introduction

Eosinophils are granulocytic white blood cells involved in health and disease. Normal levels in peripheral blood range between 0 and 0.5 × 10^9^/L and increase in various diseases [1,2].

Classically, eosinophils have been considered as end-stage effector cells, mainly characterized by the release of granule-derived cytotoxic proteins and lipids, but they also have an immunoregulatory effect on other cells [3]. The role of eosinophils in cardiovascular diseases (CVDs) is not completely understood. While an increase in count, eosinophilia, is often considered pathologic [2], a reduction in count, eosinopenia, during acute myocardial infarction (AMI) was associated with a higher incidence of heart failure (HF) and death [4,5]. 

Changes in eosinophil blood count have been reported in patients with rheumatoid arthritis receiving anakinra, a recombinant interleukin-1 (IL-1) receptor antagonist [6]. We recently described that in patients with ST-segment elevation myocardial infarction (STEMI), anakinra leads to a significant reduction in leukocyte count with a relative reduction in neutrophils and an increase in eosinophils [7]. In patients with STEMI, anakinra was also associated with a reduction in HF-related events [8,9,10,11].

Anakinra is also frequently associated with injection site reactions (ISR), characterized by erythema, inflammation, and pain. These reactions usually occur 1–2 weeks after the start of treatment, tend to be mild and transient, and eosinophils have been shown to play a role [12,13].

Whether anakinra is associated with changes in eosinophil count and whether these changes are associated with a different response in systemic inflammation (changes in C-reactive protein, CRP), cardiorespiratory fitness (CRF), cardiac systolic and diastolic function are unknown. Moreover, whether ISR represents an eosinophilic response to anakinra and is associated with a different response to treatment in patients with HF are also unknown. The aim of the study was to evaluate the role of the changes in eosinophil blood count in patients with HF treated with anakinra, and to determine whether changes in eosinophils correlate with clinical or functional parameters in patients with HF, and with the incidence of ISR.

## 2. Materials and Methods

### 2.1. Patient Population

We analyzed data from patients with HF who were treated with anakinra (Kineret^®^, Swedish Orphan Biovitrum, Waltham, MA, USA), and underwent blood sampling at baseline and during treatment. Transthoracic echocardiogram (TTE), cardiopulmonary exercise testing (CPX), and quality of life assessments were performed at each visit as well. In a portion of patients, the same assessment was repeated after discontinuation of the treatment.

The analysis included the pooled active treatment arms of four clinical trials. The pilot study of the safety and efficacy of Anakinra in Heart Failure (AIR-HF) [14] was a phase 2, open-label, single-arm pilot trial that enrolled 7 patients with HF with reduced ejection fraction (HFrEF) and elevation of high-sensitivity CRP (>2 mg/L), baseline blood sampling, and CPX, and subsequently subjected them to treatment with anakinra 100 mg daily for 14 days, with repeat testing at the end of anakinra treatment. The pilot feasibility study of the safety and efficacy of Anakinra in Heart Failure With Preserved Ejection Fraction (D-HART) [15] was a phase 2, pilot crossover trial that randomly assigned 12 patients with stable HF with preserved ejection fraction (HFpEF), and with significant symptoms (New York Heart Association [NYHA] class II or III) and baseline elevation of high-sensitivity CRP (≥2 mg/L) to receive either anakinra 100 mg daily for 14 days or placebo. Blood sampling and CPX were performed at the end of each 14-day treatment course, and 2 weeks after completing an experimental treatment course. The D-HART2 trial [16] randomized 31 patients with HFpEF and elevated baseline high-sensitivity CRP (>2 mg/L) to either anakinra 100 mg daily (*n* = 21) or placebo (*n* = 10) for 12 weeks. Blood sampling and CPX were performed at 4, 12, and 24 weeks from enrollment. Finally, the Recently Decompensated Heart Failure Anakinra Response Trial (REDHART) trial [17] enrolled patients with HF with HFrEF, and elevated CRP (>2 mg/L), within 14 days from discharge after a HF hospitalization. Patients were randomized to receive either anakinra 100 mg daily for 12 weeks (*n* = 20), anakinra 100 mg daily for 2 weeks (*n* = 20), or placebo (*n* = 20). Patients underwent blood sampling and CPX at 2, 4, 12, and 24 weeks. Patients with acute infections as well as severe asthma or chronic pulmonary obstructive diseases were excluded in the original trials. All studies were approved by the local Institutional Review Board and were conducted according to the Declaration of Helsinki and Good Clinical Practice. All patients provided written informed consent.

### 2.2. Laboratory Data

A complete blood cell count (CBC) with differential count was obtained at baseline, during treatment with anakinra, and again after discontinuation of the treatment. White blood cell count, lymphocyte, neutrophil, eosinophil, and basophil counts were calculated using a hematology analyzer. We also calculated the leukocyte-to-eosinophil ratio (LER) and the neutrophil-to-eosinophil ratio (NER), as an expression of preferential eosinophilic maturation and increase. High-sensitivity CRP, chosen as a surrogate for IL-1 activity, and N-terminal pro B-type natriuretic peptide (NT-proBNP), a biomarker of myocardial strain, were also measured at each visit. Data from the last available laboratory test while on anakinra were collected as on-treatment, while data from last available laboratory test after treatment suspension were collected as off-treatment.

### 2.3. Cardiorespiratory Fitness, Cardiac Function, and Quality of Life

A supervised maximal aerobic CPX was administered using a conservative ramping treadmill protocol, as previously described [14,15,16,17]. The peak oxygen consumption (VO_2_), minute ventilation to carbon dioxide production (VE/VCO_2_) slope, exercise time, peak respiratory exchange ratio (RER), and the oxygen uptake efficiency slope (OUES) were considered.

Furthermore, all patients underwent TTE during the same outpatient visit as the laboratory data and prior to CPX, according to the study protocols [17]. The left-ventricular ejection fraction (LVEF) and pulsed-wave Doppler early mitral flow velocity to early mitral annulus tissue velocity (E/e′) ratio were considered as LV systolic and diastolic function indices, respectively.

Symptom burden and quality of life were assessed at each visit with the Duke Activity Status Index (DASI) [18] and the Minnesota Living with Heart Failure (MLHFQ) questionnaire [19]. A lower DASI score reflects impaired perceived functional capacity, whereas a higher MLHFQ score reflects greater HF symptom burden.

### 2.4. Objective of the Analysis

The primary objective of this analysis was to evaluate changes in eosinophils in patients with HF treated with anakinra. Secondary objectives were to evaluate whether these changes are associated with the incidence of ISR and changes in CRP, CRF, and cardiac systolic and diastolic function.

### 2.5. Statistical Analyses

Individual patient data were pooled in a single prespecified data set and analyzed. Continuous variables were tested for normal distribution using the Kolmogorov–Smirnov test. Baseline characteristics were reported as number (percentage) or median [interquartile range]. The correlation between continuous variables was assessed using Spearman’s rank correlation coefficient. Categorical variables were compared using chi-square test or Fisher’s exact test, when indicated. The within-group paired differences were compared using the Wilcoxon signed-rank test. A 2-sided *p*-value of less than 0.05 was considered statistically significant. All analyses were performed using IBM SPSS Statistics 26 (IBM, Armonk, NY, USA).

## 3. Results

We identified 64 patients with HF who received anakinra treatment and had an available CBC with differential before and during anakinra treatment (on-treatment analysis). Among those, 41 (64%) also repeated the assessment after discontinuation of the treatment (off-treatment analysis). The median age was 55 (51–63) years, and 32 (50%) were biological females and 47 (73%) self-identified as Black/African-American. Baseline CRP was 6.2 (3.0–15.4) mg/L, LVEF was 49 (33–60)%, E/e’ was 14.2 (9.7–19.9), peak VO_2_ was 14.2 (11.6–16.9) mLO_2_·kg^−1^·min^−1^, and the VE/VCO_2_ slope was 31 (27–35). The duration of on-treatment with anakinra was 4 (2–12) weeks, whereas the time from discontinuation of anakinra to the latest off-treatment assessment was 12 (4–12) weeks. Baseline demographic and clinical characteristics are summarized in Table 1. There were no statistically significant differences in clinical characteristics between the patients with on-treatment analysis and those with off-treatment analysis (all *p* > 0.05).

**Figure 1:**
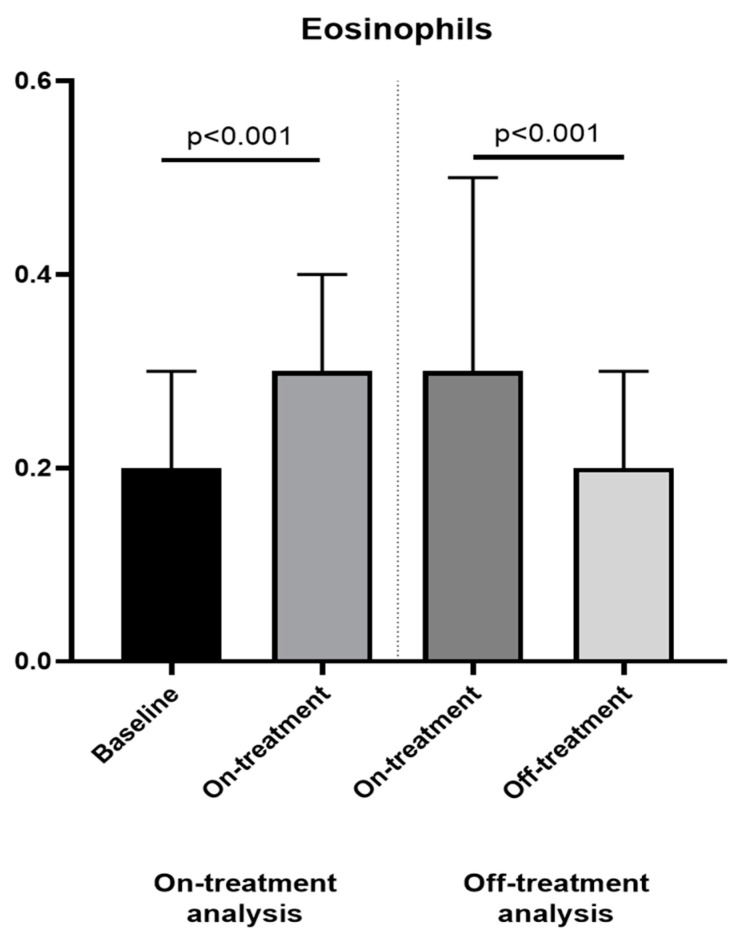
Changes in eosinophils between baseline, on and off-treatment analyses. We found a significant change in eosinophils between baseline and on-treatment (on-treatment analysis, **left** panel) and between on and off-treatment (off-treatment analysis, **right** panel).

Treatment with anakinra was associated with a significant increase in eosinophils (from 0.2 [0.1–0.3] × 10^3^ cells/µL to 0.3 [0.1–0.4] × 10^3^ cells/µL, *p* < 0.001) and this change was reverted after the discontinuation of the treatment (from 0.3 [0.2–0.5] × 10^3^ cells/µL to 0.2 [0.1–0.3] × 10^3^ cells/µL, *p* < 0.001), as shown in Figure 1. A decrease in NER (from 21.0 [14.3–36.8] to 9.5 [6.4–20.9], *p* < 0.001) and LER (from 38.0 [21.7–56.0] to 19.0 [12.9–40.1], *p* = 0.001) during treatment was observed, and this was reversed after suspension (from 8.0 [5.3–13.0] to 18.5 [10.7–35.0] for NER, *p* < 0.001 and from 16.0 [11.5–27.7] to 31.0 [19.5–64.0] for LER, *p* < 0.001).

**Figure 2:**
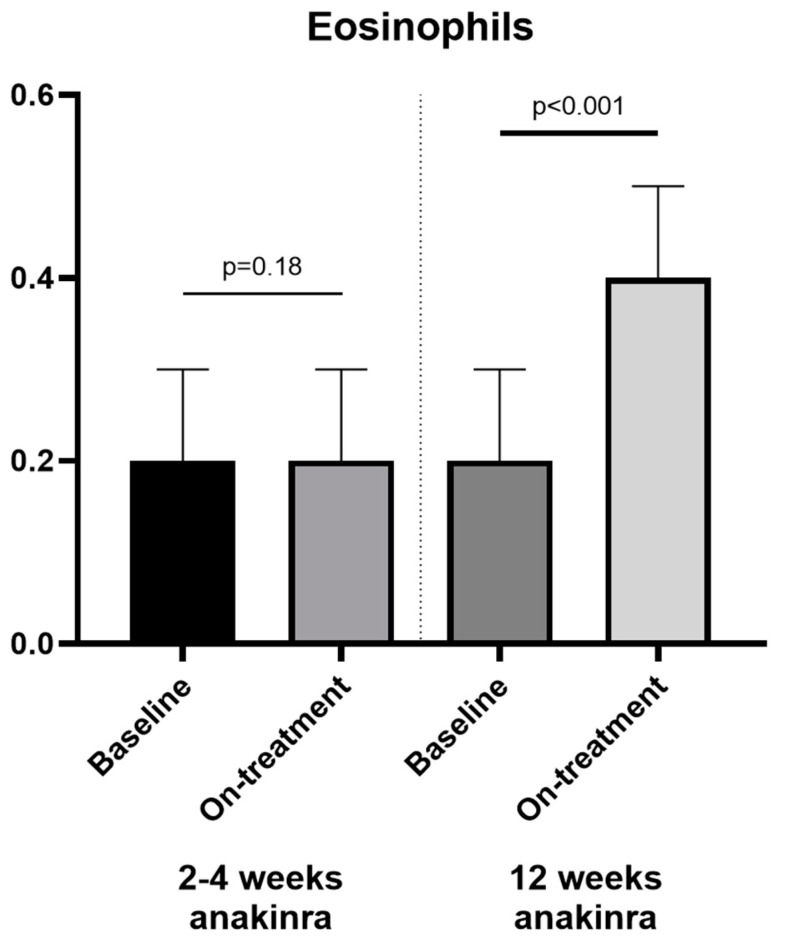
Duration of anakinra treatment and changes in eosinophils. We found a significant change in eosinophils between baseline and on-treatment analysis in the patients who received anakinra for 12 weeks (**right** panel) and not in the 2–4 weeks treatment (**left** panel).

Comparing the patients treated for 2–4 weeks (N = 35) and patients treated for 12 weeks (N = 29), we found no significant changes in eosinophils in patients treated with anakinra for 2–4 weeks (from 0.2 [0.1–0.3] × 10^3^ cells/µL to 0.2 [0.1–0.3] × 10^3^ cells/µL, *p* = 0.18), and a statistically significant increase in eosinophils (from 0.2 [0.1–0.3] × 10^3^ cells/µL to 0.4 [0.2–0.5] × 10^3^ cells/µL, *p* < 0.001) in patients treated with anakinra for 12 weeks (absolute change in eosinophil was 0 [0–0.1] × 10^3^ cells/µL and 0.2 [0–0.3] × 10^3^ cells/µL, for patients treated for 2–4 and 12 weeks, respectively, *p* < 0.01) (Figure 2). The duration of treatment did not correlate with the changes in peak VO_2_. These data show a greater increase in eosinophils with longer duration of treatment.

Of note, the changes in eosinophils over time were significantly positively correlated with the changes in peak VO_2_ (Spearman’s Rho = +0.228, *p* = 0.020), DASI score (Rho = +0.261, *p* = 0.015), and negatively with changes in CRP (Rho = −0.297, *p* = 0.002), NT-proBNP (Rho = −0.222, *p* = 0.041), and the E/e′ ratio (Rho = −0.288, *p* = 0.011). No significant correlations were found for changes in the other parameters (VE/VCO_2_ slope, Rho = −0.040, *p* = 0.690; exercise time, Rho = +0.185, *p* = 0.059; RER, Rho = +0.160, *p* = 0.11; OUES, Rho = +0.069, *p* = 0.849; LVEF, Rho = −0.029, *p* = 0.795; MLHFQ, Rho = −0.132, *p* = 0.227), as shown in Table 2.

Both changes in NER and LER correlated negatively with the changes in peak VO_2_ (Rho = −0.303, *p* = 0.002 and Rho = −0.344, *p* < 0.001, respectively) and DASI sore (Rho = −0.358, *p* = 0.001 and Rho = −0.360, *p* = 0.001, respectively), and positively with changes in CRP (Rho = +0.525, *p* < 0.001 and Rho = +0.504, *p* < 0.001, respectively). On the other hand, only LER correlated with MLHFQ score (Rho = +0.251, *p* = 0.026).

**Figure 3:**
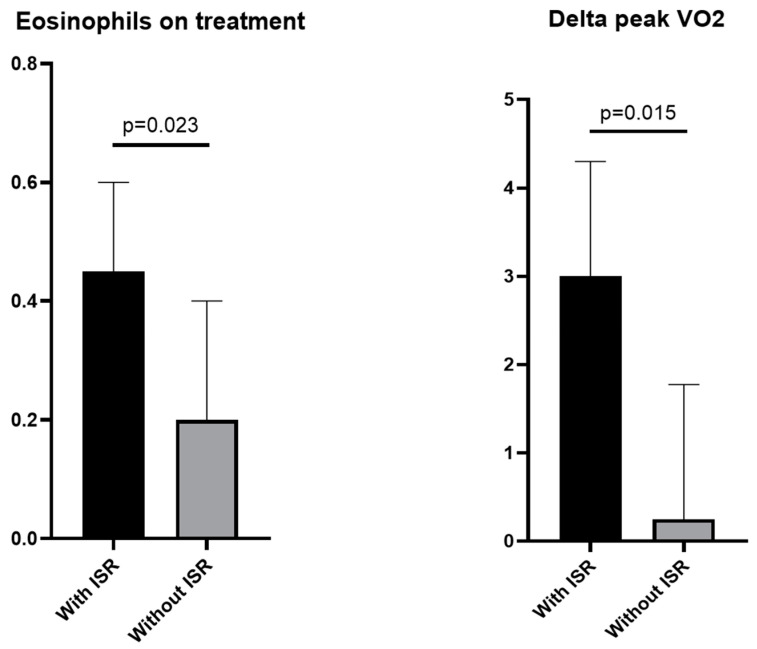
Injection site reactions, eosinophils, and peak VO_2_ changes. We found a statistically significant difference in eosinophils (**left** panel) and change (delta) in peak VO_2_ (**right** panel) between patients who experienced and did not experience injection site reactions. Abbreviation: ISR, injection-site reactions.

ISR were reported by eight (13%) patients (median age of 51 [45–59] years), of which four (50%) self-identified as females and four (50%) as Black/African-American. None of these ISR patients required drug discontinuation. There was no significant difference between patients with or without ISR at baseline; on the other hand, patients with ISR had higher on-treatment eosinophil counts (0.45 [0.38–0.60] × 10^3^ cells/µL vs. 0.20 [0.10–0.40] × 10^3^ cells/µL, *p* = 0.023), higher absolute peak VO_2_ (18.5 [14.5–25.2] mLO_2_·kg^−1^·min^−1^ vs. 14.9 [12.3–17.2] mLO_2_·kg^−1^·min^−1^, *p* = 0.044), larger change in peak VO_2_ (3.0 [0.9–4.3] mLO_2_·kg^−1^·min^−1^ vs. 0.3 [−0.6–1.8] mLO_2_·kg^−1^·min^−1^, *p* = 0.015), longer on-treatment exercise time (650 [490–830] seconds vs. 500 [370–583] seconds, *p* = 0.016), and lower on-treatment NT-proBNP levels (81 [13–120] pg/mL vs. 445 [95–1187] pg/mL, *p* = 0.003), as shown in Table 3 and Figure 3.

## 4. Discussion

We herein report, for the first time, that IL-1 blockade with anakinra leads to a significant transient increase in eosinophils in patients with established HF, with greater changes with longer duration. These changes are reversed after discontinuation of treatment. Moreover, the changes in eosinophils were inversely related to markers of systemic inflammation (i.e., CRP) and directly related to greater cardiorespiratory fitness (peak VO_2_).

Eosinophilia after treatment with anakinra has been reported in as many as 9% of patients with rheumatoid arthritis [6], although the exact mechanism and function is unknown. We recently reported an increase in eosinophils in patients with STEMI treated with anakinra [7] in randomized clinical trials associated with a reduction in HF events. The effects of anakinra on eosinophils, and the implications of this in patients with HF treated with anakinra are, however, rather unexplored.

Eosinophilia is associated with severe cardiovascular manifestations such as endocarditis and thrombosis, endomyocardial fibrosis, and valvular disease [2]. The elevation of eosinophils, even if milder, has been associated with coronary artery disease prevalence [20]. On the other side, eosinophils have also recently been shown to have a cardioprotective role in preclinical models of AMI [21], protecting cardiomyocytes from ischemia injury, reducing cardiomyocyte death, and regulating cardiac fibroblast activity and post-AMI inflammatory cell adhesion and infiltration. Eosinopenia, a reduction in eosinophils, has been reported as a surrogate of hyper-inflammation, linked to adverse cardiac remodeling and function following AMI [22], and associated with worse clinical outcomes over long-term follow-up [4,5]. Vural A et al. [23] showed that NER and LER are predictive for 6-month mortality and major adverse cardiovascular events in patients with acute decompensated HF with reduced EF. On this basis, the increased eosinophil counts observed during treatment with anakinra in STEMI patients [7] could suggest a way by which the drug modulates the innate immune inflammatory response and prevents post-AMI HF events. We herein show a significant reduction in NER and LER during treatment with anakinra, which is reversed after suspension.

Injection-site reaction during treatment with anakinra is the most common side effect, reported in more than 70% of cases in some series, and this finding can be associated with an increase in circulating eosinophils and dermal infiltrate of eosinophils [13]. We showed for the first time that patients with ISR had increased eosinophil counts but also a greater response in CRF (greater improvements in peak VO_2_), supporting the concept that an enhanced eosinophilic response to anakinra is associated with clinical improvement. Accordingly, we also found a positive correlation between eosinophils, NER, LER, and the changes in peak VO_2_ and perceived functional capacity (DASI score). Whether the difference in improvement in CRF was dependent on the different baseline characteristics between patients with or without injection site reactions, or whether it was a biomarker of a greater response to the treatment cannot be determined with the current study and requires additional research. Moreover, whether the change in eosinophils is simply a biomarker or also a mediator of the anti-inflammatory mechanism of anakinra is not known. The inverse correlation between eosinophils and measures of impaired cardiac diastolic function (NT-proBNP, and E/e′ ratio) and the direct correlation with cardiorespiratory fitness (peak VO_2_) suggest a possible cardioprotective effect of eosinophils.

## 5. Conclusions

Patients with HF treated with anakinra experience a transient increase in blood eosinophil counts. The eosinophil count may serve as a favorable prognostic biomarker for IL-1 blockade in HF, and patients with ISR may represent a subset of patients with an enhanced eosinophilic response that experience a greater benefit from the treatment. Furthermore, these data may also open the way to new mechanistic research about the roles of IL-1 and eosinophils in HF.

## Figures and Tables

**Table 1:** Clinical characteristics of patients in the on-treatment and off-treatment analyses.

	On-Treatment Analysis (N = 64)			Off-Treatment Analysis (N = 41)		
Clinical variables						
Age (years)	55 [51–63]			54 [51–59]		
Sex—F (%)/M (%)	32 (50)/32 (50)			20 (49)/21 (51)		
Race—Black (%)/White (%)	47 (73)/17 (27)			30 (73)/11 (27)		
BMI (kg/m^2^)	38 [32–44]			38 [34–44]		
NYHA class						
NYHA class II	24 (38)			20 (49)		
NYHA class III	40 (63)			21 (51)		
CAD	15 (23)			9 (22)		
DM	38 (59)			22 (54)		
HTN	58 (90)			35 (85)		
HLP	45 (66)			28 (68)		
ACEi/ARB	49 (77)			32 (78)		
Beta-blocker	57 (89)			37 (90)		
MRA	30 (47)			22 (54)		
Biomarkers	Baseline	On-Treatment	*p*	On-treatment	Off-Treatment	*p*
WBC (×10^3^ cells/µL)	6.5 [5.6–8.2]	5.8 [4.5–6.9]	<0.001	5.7 [4.7–7.0]	6.6 [5.4–8.2]	<0.001
Lymphocytes (×10^3^ cells/µL)	1.9 [1.6–2.3]	1.9 [1.6–2.4]	0.322	1.9 [1.6–2.4]	1.9 [1.6–2.2]	0.420
Neutrophils (×10^3^ cells/µL)	4.1 [3.2–5.5]	2.9 [2.0–4.0]	<0.001	2.9 [2.0–4.0]	4.1 [2.9–5.4]	<0.001
Eosinophils (×10^3^ cells/µL)	0.2 [0.1–0.3]	0.3 [0.1–0.4]	<0.001	0.3 [0.2–0.5]	0.2 [0.1–0.3]	<0.001
Basophils (×10^3^ cells/µL)	0.1 [0–0.1]	0 [0–0.1]	<0.028	0 [0–0.1]	0.1 [0–0.1]	0.285
Neutrophil-to-eosinophil ratio (NER)	21.0 [14.3–36.8]	9.7 [6.4–20.9]	<0.001	8.0 [5.3–13.0]	18.5 [10.7–35.0]	<0.001
Leukocyte-to-eosinophil ratio (LER)	38.0 [21.7–56.0]	20.5 [12.9–41.6]	<0.001	16.0 [11.5–27.7]	31.0 [19.5–64.0]	<0.001

Data are expressed as median [interquartile range] or number (%). Abbreviations: F, female; M, male; AA, African-American; BMI, body mass index; CAD, coronary artery disease; DM, diabetes mellitus; HTN, hypertension; HLP, hyperlipidemia; ACEi, angiotensin converting enzyme inhibitor; ARB, angiotensin II receptor blockade; MRA, mineralocorticoid receptor antagonist; CRP, C-reactive protein; NT-proBNP, N-terminal pro b-type natriuretic peptide.

**Table 2:** Correlations between changes in eosinophils and peak VO_2_, DASI score, C-reactive protein, NT-proBNP, and E/e′ in patients with heart failure treated with anakinra.

Variables		C-Reactive Protein	Peak VO_2_	VE/VCO_2_ Slope	Exercise Time	RER	OUES	NT-proBNP	LVEF	E/e′	DASI Score	MLHFQ
Changes in eosinophils	Spearman’s Rho	−0.297	+0.228	−0.040	+0.185	+0.160	+0.069	−0.222	−0.029	−0.288	+0.261	−0.132
	*p value*	0.002	0.020	0.690	0.059	0.11	0.849	0.041	0.795	0.011	0.015	0.227

Abbreviations: VE/VCO_2_, minute ventilation/carbon dioxide production; RER, respiratory exchange ratio; OUES, oxygen uptake efficiency slope; NT-proBNP, N-terminal pro b-type natriuretic peptide. LVEF, left ventricle ejection fraction; DASI, Duke Activity Status Index; MLHFQ, Minnesota Living with Heart Failure Questionnaire.

**Table 3:** Clinical characteristics, cardiorespiratory fitness, and biomarkers in patients with and without injection site reactions.

	Patients with Injection Site Reactions (N = 8)	Patients without Injection Site Reactions (N = 56)	*p* Value
Variables			
Age (years)	51 [45–59]	55 [52–64]	0.10
Sex—F (%)/M (%)	4 (50)/4 (50)	28 (50)/28 (50)	1.00
Race—Black (%)/White (%)	4 (50)/4 (50)	393 (70)/17 (30)	0.11
BMI (kg/m^2^)	36 [35–41]	38 [32–45]	0.45
NYHA class			
NYHA class II	4 (50)	20 (36)	0.43
NYHA class III	4 (50)	36 (64)	0.43
CAD	2 (25)	13 (23)	0.91
DM	6 (75)	32 (57)	0.34
HTN	6 (75)	52 (93)	0.11
HLP	4 (50)	41 (73)	0.18
ACEi/ARB	7 (88)	42 (75)	0.44
Beta-blocker	8 (100)	49 (88)	0.29
MRA	6 (75)	24 (43)	0.09
CPX			
Peak VO_2_ (mLO_2_·kg^−1^·min^−1^)			
- Baseline	16.8 [13.2–19.4]	14.0 [11.6–16.5]	0.16
- On-treatment	18.5 [14.5–25.2]	14.9 [12.3–17.2]	0.044
- Off-treatment	15.0 [13.7–25.5]	14.8 [11.4–17.5]	0.19
- Delta baseline-on treatment	3.0 [0.9–4.3]	0.3 [−0.6–1.8]	0.015
- Delta on-off treatment	−1.5 [−2.1–0.7]	−0.3 [−2.1–0.6]	0.62
Exercise time (seconds)			
- Baseline	595 [505–725]	455 [290–548]	0.007
- On-treatment	650 [490–830]	500 [370–583]	0.016
- Off-treatment	730 [580–870]	510 [375–630]	0.026
Biomarkers			
CRP (mg/L)			
- Baseline	2.8 [2.3–13.3]	6.5 [3.6–15.5]	0.12
- On-treatment	1.3 [0.5–7.2]	1.4 [0.9–3.7]	0.81
- Off-treatment	2.1 [0.6–9.7]	6.8 [2.9–13.6]	0.11
NT-proBNP (pg/mL)			
- Baseline	74 [13–219]	634 [200–1774]	0.002
- On-treatment	81 [13–120]	445 [95–1187]	0.003
- Off-treatment	57 [20–120]	383 [121–1094]	0.001
WBC (×10^3^ cells/µL)			
- Baseline	6.1 [5.2–7.8]	6.6 [5.6–8.3]	0.50
- On-treatment	6.1 [4.4–7.4]	5.6 [4.5–6.7]	0.44
- Off-treatment	6.2 [5.4–7.3]	6.9 [5.4–8.3]	0.48
Neutrophils (×10^3^ cells/µL)			
- Baseline	3.5 [2.5–5.1]	4.4 [3.2–5.7]	0.27
- On-treatment	2.9 [1.9–5.1]	2.9 [2.0–4.0]	0.75
- Off-treatment	3.7 [2.7–4.3]	4.4 [3.1–5.7]	0.34
Eosinophils (×10^3^ cells/µL)			
- Baseline	0.3 [0.1–0.4]	0.2 [0.1–0.3]	0.21
- On-treatment	0.5 [0.3–0.6]	0.2 [0.1–0.4]	0.023
- Off-treatment	0.2 [0.2–0.4]	0.2 [0.1–0.3]	0.34

Data are expressed as median [interquartile range] or number (%). Abbreviations: F, female; M, male; AA, African-American; BMI, body mass index; CAD, coronary artery disease; DM, diabetes mellitus; HTN, hypertension; HLP, hyperlipidemia; ACEi, angiotensin converting enzyme inhibitor; ARB, angiotensin II receptor blockade; MRA, mineralocorticoid receptor antagonist; LVEF, left ventricular ejection fraction; CPX, cardiopulmonary exercise test; VO_2_, oxygen consumption; CRP, C-reactive protein; NT-proBNP, N-terminal pro b-type natriuretic peptide.

## Data Availability

Data are available on request. Outcome is dependent upon review from the Institutional Review Board.
